# Effect of moxibustion on meridian in a warm needling model: A protocol for a prospective observational study

**DOI:** 10.1097/MD.0000000000031492

**Published:** 2022-11-25

**Authors:** Chiao-Hsuan Hsieh, Shih-Ting Tseng, Yu-Chiang Hung, Ting-Chang Chang, Wen-Long Hu, Chien-Hung Lin

**Affiliations:** a Department of Chinese Medicine, Kaohsiung Chang Gung Memorial Hospital and Chang Gung University College of Medicine, Kaohsiung, Taiwan; b Department of Physics, National Sun Yat-Sen University, Kaohsiung, Taiwan; c College of Nursing, Fooyin University, Kaohsiung, Taiwan; d Kaohsiung Medical University College of Medicine, Kaohsiung, Taiwan.

**Keywords:** acupuncture, Chinese medicine, electric characteristic, meridian theory, moxibustion, warm needling

## Abstract

Warm needling is a type of traditional Chinese medicine (TCM), which uses burned moxa to generate warmth and exerts a therapeutic effect on human skin. Additionally, based on TCM theory, warm needling could improve deficiency-cold syndrome. Research has found that warm needling has analgesic effects as well as effects on the immune system, endocrine system, nervous system, and lower urinary tract symptoms. However, the mechanism of warm needling on the meridians is not fully understood. In modern studies, the electric characteristics of the meridians have been discovered and the combination of semiconductor and TCM theory could explain the meridian phenomena through physics. However, there is no known research exploring the relationship between thermal effect and electric property on the meridians.

**Methods:**

A total of 30 healthy participants older than 20 years of age will be recruited for this study. The participants would first be administered acupuncture, following which the electric characteristics will be measured using the semiconductor analyzer Agilent B1500A/Agilent 4156C. The visual analog score (VAS) will also be rated. Thereafter, a burned moxa will be added to the acupuncture needles as a method for warm needling. The electric characteristics and VAS will be measured again. We will use the paired t-test and repeated measure two-way ANOVA to compare the electric characteristics and VAS before and after warm needling in each participant.

**Objectives:**

This protocol aims to explore the thermal effect on the electric characteristics of meridians in a warm needling model and provide a scientific explanation of TCM through the aspect of physics.

## 1. Introduction

Acupuncture has been widely used to treat various diseases and relieve pain.^[1]^ In traditional Chinese medicine (TCM) theory, there are some methods to improve the therapeutic effects of acupuncture, including manual manipulation, electroacupuncture (EA), moxibustion, or warm needling.^[1–4]^ Warm needling is a combination therapy of acupuncture and moxibustion by placing an ignited moxa on the handle of an acupuncture needle.^[5]^ This could stimulate acupoints by needling, heating, and pharmacological effect of moxa.^[4]^ It is thought that warm needling could warm meridians and improve deficiency-cold syndrome.^[6]^ Modern research has found that warm needling has effects on the immune, nervous, and endocrine systems^[7,8]^ and that it relieves pain^[5]^ and improves the range of motion of the shoulders,^[9]^ knee osteoarthritis,^[6]^ and lower urinary tract symptoms,.^[4]^ However, how the warm needling works on the meridians has not been identified yet.^[6]^

There is no definitive conclusion regarding what meridians and acupoints essentially are.^[10]^ Many researchers have been exploring several ways, including biophysics and biochemistry, to elucidate the meridians.^[11]^ Researchers explored meridians and acupoints according to the physical and electric characteristics of the human skin, and the result showed that some specific parts of the skin had the characteristic of higher conductivity, lower resistance, and higher potential.^[12,13]^ Furthermore, the researchers found that the specific parts of the skin and acupoints on the meridians were matched.^[14–16]^ Recently, some studies combined semiconductor and TCM theory to discuss the meridian phenomena and the relationship between electric characteristics and meridians.^[17,18]^ Hung et al introduced the ion current model and compared different kinds of pulses, and found that ions might be the substance delivered by the meridians.^[17]^ Wang et al applied supercritical fluid technology to discriminate the relation between different conductivity of acupuncture needles and the electric properties of the meridians. It was proven that electrical conductivity and the therapeutic effects of the meridian could be improved by supercritical fluid technology.^[18]^ Additionally, the directions of ion transport in the meridians and changing the direction of needling could influence the electricity of the meridians.^[19]^ Moreover, the change of needling direction could also affect the electric current, which implies that the acupuncture manipulations might produce different effects in humans.^[20]^

The change in the physical properties of the acupuncture needle could produce different electric characteristics in the meridians and the phenomena could be explained by physics.^[17–20]^ However, there is no published research on the relationship between temperature and electric characteristics in the meridians. The purpose of our study is to combine the theory of TCM and physics to explore the thermal effect on the electric characteristics of meridians in a warm needling model. We believe that our study can provide more scientific information about the effect of temperature on the meridians in physics.

## 2. Material and methods

### 2.1. Study design

This is a prospective and observational study that will be conducted by the Department of Chinese Medicine at the Kaohsiung Chang Gung Memorial Hospital and the Department of Physics at National Sun Yat-sen University from March 2022 to December 2022. The participants will be recruited from the students and staff at National Sun Yat-sen University. Our study will enroll 30 healthy participants in 1 group and all of them will receive both interventions, acupuncture and warm needling, and electric measurement. All personal data will be preserved in a locked closet and kept confidential until the end of the experiment when the principal investigator will disclose and analyze the results.

**Figure 1.**
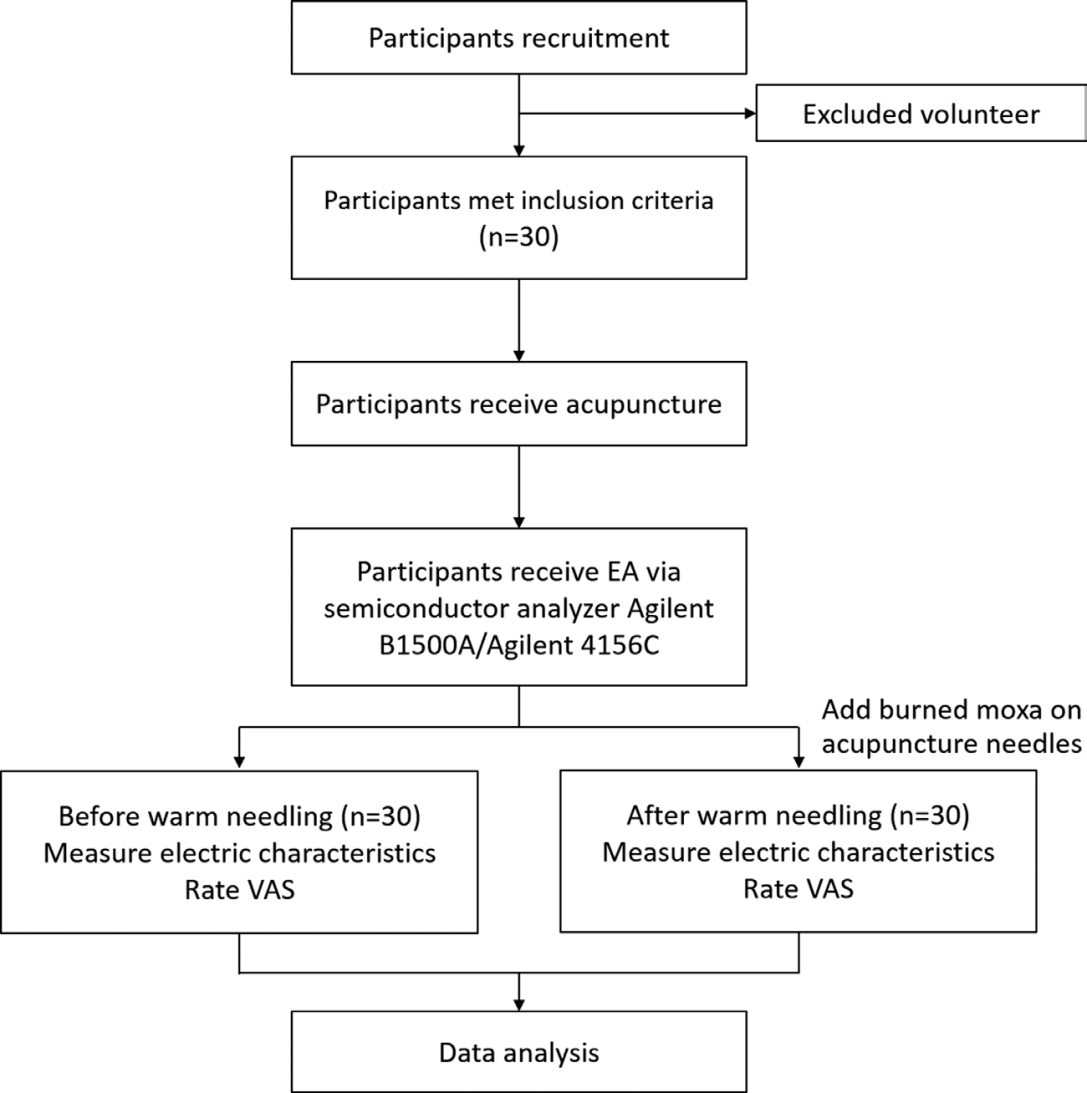
Flowchart of the study design. EA = electroacupuncture, VAS = visual analog scale.

The participants will receive acupuncture, following which we will use a semiconductor analyzer to measure the electric characteristics and evaluate the visual analog scale (VAS) score before warm needling. Subsequently, we will place burned moxa on the acupuncture needles as our warm needling method. After the moxa burns out, we will measure the electric characteristics using the same evaluation method. We will analyze 1 meridian at a time and measure 12 meridians sequentially in this study. Therefore, all participants will undergo 12 sessions of measurements. Finally, we will compare individual electric characteristics data, before and after warm needling, on 12 meridians. The measurements will be completed in 1 day and the participants would not have to return for follow-up. The flow chart of the study design is presented in Figure 1.

### 2.2. Participants

The participants who are healthy, aged over 20 years, and willing to sign the informed consent will be considered eligible for the study. The exclusion criteria for this study are: aged below 20 years, pregnant or breast-feeding women, empty stomach before the study, bleeding tendency due to thrombocytopenia or platelets <20,000/µL or taking antiplatelet medicine, and consuming hypnotics or psychoactive medicines. We intend to recruit 30 participants for this study.

### 2.3. Interventions

**Figure 2.**
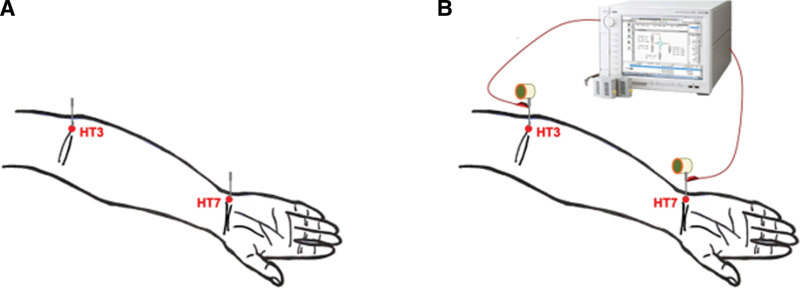
(A) Acupuncture on 2 acupoints of one meridian. (B) Perform EA and measure electric characteristics using semiconductor analyzer Agilent B1500A/Agilent 4156C; subsequently, add the burned moxa on the acupuncture needles as warm needling. EA = electroacupuncture.

All the participants in our study will receive acupuncture and warm needling. The acupuncture needles will be sterile, disposable, and 40 mm in length and 0.3 mm in diameter or 25 mm in length and 0.3 mm in diameter. The acupuncture needles would be obtained from the same manufacturer (COSMOS INTERNATIONAL SUPPLIES CO., LTD.). We will perform acupuncture on 12 meridians and choose 2 acupoints in each meridian (Fig. 2A).

We will add the burned moxa to the handle of the acupuncture needles as a method for warm needling (Fig. 2B). The moxa rolls are made with pure moxa and rolled in rice paper. The moxa rolls will be 1.2 cm in diameter, 1.5 cm long, and weigh about 0.9 g each. The moxa rolls would be obtained from the same manufacturer (MAC Co.). It will take about 5 to 10 minutes for the moxa to burn out.

**Figure 3.**
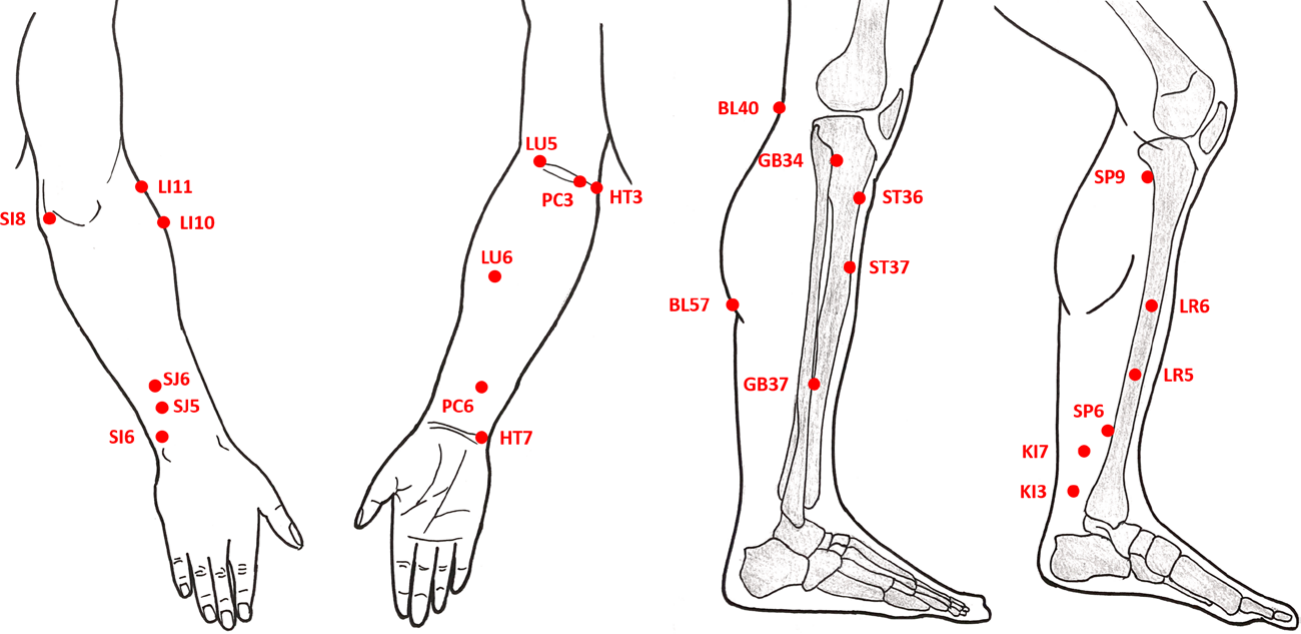
The schematic picture of acupoints in our study.

The acupoints would be: Chize (LU5) and Kongzui (LU6), Shousanli (LI10) and Quchi (LI11), Zusanli (ST36) and Shangjuxu (ST37), Sanyinjiao (SP6) and yinlingquan (SP9), Shaohai (HT3) and Shenmen (HT7), Yanglao (SI6) and Xiaohai (SI8), Weizhong (BL40) and Chengshan (BL57), Taixi (KI3) and Fuliu (KI7), Quze (PC3) and Neiguan (PC6), Waiguan (SJ5) and Zhigou (SJ6), Yanglingquan (GB34) and Guangming (GB37), and Ligou (LR5) and Zhongdu (LR6). The schematic picture of acupoints lists in Figure 3. These acupoints are based on the World Health Organization standard acupuncture point locations.^[21]^

### 2.4. Outcome measurement

The primary outcomes are the electric characteristics of each meridian measured using the semiconductor analyzer Agilent B1500A/Agilent 4156C, which has been used for meridian electricity evaluation.^[20]^ This machine integrates multiple measurements, including fundamental current-voltage (IV), capacitance-voltage, pulsed IV measurement, and analysis capabilities. The IV measurement ranges from 0.1 fA to 1 A and 0.5 µV to 200 V. The maximal pulse forcing is 40 V and the minimal sampling interval is 5 ns.^[17]^ We would adjust the output electrical parameters. Further, the voltage and current will be supplied by the semiconductor analyzer Agilent B1500A/Agilent 4156C and the electric characteristics will be measured immediately as baseline data (before warm needling) (Fig. 2B). After the moxa burns out, we will again measure the electric characteristics (after warm needling).

The secondary outcome is VAS, a tool for pain assessment. The VAS is a self-reported pain rating scale recorded between 0 and 10.^[22]^ A lower score means less pain while a higher score means more pain. VAS has been used to rate the common *De Qi* sensations subjectively, including ache, numbness, heaviness, pressure, and twinge.^[23]^ Hence, we assess the *De Qi* sensations before and after warm needling by VAS.

### 2.5. Sample size calculation

The effect indicators of our study are the electric characteristic and VAS score of *De Qi* sensation before and after warm needling. Based on the results of the previous experiment, which applied the semiconductor analyzer to 30 healthy individuals, the different electric characteristic of meridians was obtained by different input direction on the meridians.^[19]^ The electric currents in different directions were 0.49 ± 0.11 and 0.38 ± 0.13 in the Lung meridian.^[19]^ Another study showed that the VAS score of *De Qi* sensations in healthy participants were 1.54 ± 1.62 and 1.87 ± 1.88 in the traditional needle group and special needle group, respectively.^[18]^ Thus, we used G*Power 3.0.1.0 software to calculate our sample size, with power = 0.8, alpha = 0.05, effect size convention *R* = 0.53. The required sample size of each group was calculated to be 30 participants.

### 2.6. Statistical analysis

Paired *t* test and repeated measure two-way ANOVA will be used to analyze the change of electric characteristics and VAS between baseline data and post-warm needling data of each participant. We set the statistical significance at a *P* value of < .05. Our statistical analyses will be conducted using SPSS for Windows, version 22 (Statistics 22, SPSS, IBM Corp., Chicago, IL).

### 2.7. Data monitoring

Acupuncture, EA, and warm needling are general practices in TCM. Minor adverse side effects of acupuncture and EA include minor bruising, bleeding or pain at needle insertion sites,^[24]^ nausea, sweating, and dizziness.^[25]^ Other complications include pneumothorax and septicemia. Most adverse side effects are minor, and serious complications are rare.^[25]^ The most common adverse events related to warm needling are burns, skin rash, and pain.^[7]^ Warm needling therapy has been reported as a safe method in clinical practices.^[4]^ In our study, acupuncture will be performed by qualified doctors and the acupoints we chose are at the 4 limbs. The procedure will be safe with minimal risk of serious adverse events. The device we use for EA will apply voltage from 1 to 60 V and current under 2 mA. The voltage and current are in the therapeutic range, which means that our study is safe. Additionally, we will perform warm needling, which adds moxa on acupuncture needles and introduces heat stimulation through the needles, and thick cardboard will be placed on the skin surface to prevent burning injury. If serious adverse effects of acupuncture occur, such as fainting or general weakness, the doctor will remove the acupuncture needles and stop the intervention immediately, and we will record the reason for dropping out of the study.

## 3. Discussion

 Some reviews of animal models discussed the mechanism of warm needling; however, most of these investigated molecular biological mechanisms. Warm needling regulated cytokines such as interleukin-1β,^[26]^ tumor necrosis factor-α,^[27]^ and transforming growth factor-β1^[28]^ to inhibit the inflammatory response. Warm needling also inhibited the overexpression of insulin-like growth factor-1 to achieve the effects of improving pathological changes in the tissue.^[28]^ Besides, warm needling modulated nicotinamide adenine dinucleotide phosphate oxidase 2 and superoxide dismutase 2, which are associated with oxidative stress.^[26]^ Chondrocyte cytoskeletal proteins such as Rho-associated protein kinase, phospho-cofilin, and monopherine domain kinase 1 were down-regulated by warm needling, which relieved symptoms of osteoarthritis.^[29]^ Histopathological changes observed using transmission electron microscopy showed an increase in the number of organelles with rearrangement and redistribution of the extracellular matrix after intervention with warm needling.^[30]^ Moreover, warm needling down-regulated matrix metalloproteinase-3, thereby influencing tissue remodeling.^[27]^ However, few studies are present on physical characteristics related to meridians and warm needling. In the present study, we will introduce physical parameters to measure the meridians and explore warm needling through physical perspectives.

According to the World Health Organization International Standard Terminologies On Traditional Medicine In The Western Pacific Region, warm needling is a practice of acupuncture in which the needle is warmed during needling by placing a burned moxa stick on the handle of the needle after insertion.^[31]^ This therapeutic technique delivers heat at acupoints to stimulate the circulation of Qi and blood. In TCM theory, this procedure achieves the effect of a warm supplement, which means to activate and restore a decreased function to normal.^[31]^ In the present study, EA, through the semiconductor analyzer Agilent B1500A/Agilent 4156C, applied the same voltage before and after warm needling. Recent meridian studies showed that the meridian has electric characteristics and physical properties.^[17]^ Additionally, improving the conductivity of the meridians could cause higher electric current and lower impedance in the meridians, which could enhance de-qi sensations and increase the therapeutic effect.^[32]^ Since there are different acupuncture manipulations in traditional Chinese theory, a previous study revealed that the electric current measured during acupuncture was significantly larger in supplementation manipulation than in draining manipulation in the meridians.^[20]^ Accordingly, we expected the changing electric characteristics to a higher current after warm needling would be comparable with the current measured before warm needling since warm needling is a supplementation method. There will be new physical discoveries of warm needling, acupuncture plus moxibustion, in our study.

Although the therapeutic effects of warm needling have been affirmed clinically, the mechanism of warm needling has not been widely elucidated. Few studies discussed the association between ionic conductivity and change of temperature in the biophysical field. This is an interdisciplinary study that has not been performed before, whereby TCM professionals cooperated with physics professionals, toward exploring warm needling using new methods and interventions. The results of electric characteristics related to warm needling will give a new perspective on this popular and effective TCM treatment. The electrical intervention and electric characteristics of warm needling are expected to have vast applications in medical treatment.

## 4. Ethics and dissemination

This protocol was approved by the Human Ethics Committee of Chang Gung Medical Foundation Institutional Review Board, IRB No. 202101924A3. The protocol has been registered at ClinicalTrials.gov (Identifier: NCT05249010) and ISRCTN.com (Identifier: ISRCTN82061181). All participants will provide written informed consent before enrollment. Personal information about potential and enrolled participants will be collected, shared, and maintained in an independent closet to protect confidentiality before, during, and after the trial. Data will be available under reasonable request.

## Acknowledgements

The authors would like to express our thanks to the other members of the research team of this research protocol: Yung-Fang Tan, Wen-Chung Chen, Chuan-Wei Kuo, and Chia-Chuan Wu.

## Author contributions

C.H. Lin and T.C. Chang were responsible for the design, conception, and plan of the study. C.H. Hsieh drafted the manuscript. C.H. Lin and Y.C. Hung designed the statistical plan. C.H. Lin, S.T. Tseng, and W.L. Hu participated in the revision of the manuscript and coordination of the study. All authors read, approved the final manuscript, and agreed with the submission.

**Conceptualization:** Shih-Ting Tseng.

**Investigation:** Ting-Chang Chang.

**Methodology:** Yu-Chiang Hung, Ting-Chang Chang.

**Project administration:** Yu-Chiang Hung.

**Resources:** Ting-Chang Chang.

**Software:** Ting-Chang Chang.

**Supervision:** Shih-Ting Tseng, Yu-Chiang Hung, Wen-Long Hu, Chien-Hung Lin.

**Visualization:** Wen-Long Hu.

**Writing – original draft:** Chiao-Hsuan Hsieh.

**Writing – review & editing:** Chien-Hung Lin.
